# Recent Advances in Asymmetric Catalysis Associated with B(C_6_F_5_)_3_

**DOI:** 10.3390/molecules28020642

**Published:** 2023-01-08

**Authors:** Ziye Zhan, Jiale Yan, Zhiyou Yu, Lei Shi

**Affiliations:** School of Science, Harbin Institute of Technology, Shenzhen 518055, China

**Keywords:** asymmetric catalysis, B(C_6_F_5_)_3_, Lewis acids

## Abstract

The prevalence and significance of asymmetric catalysis in the modern medicinal industry has been witnessed in recent years, which have already been used to manufacture the (S)-Naproxen and the (S)-Propranolol. With matched specificities such as the Lewis acidity and steric bulk, B(C_6_F_5_)_3_ has gained accelerating attention on its application in asymmetric catalysis of Diels–Alder cycloaddition reactions, carbonyl-ene cyclization, and other various reactions, which have been demonstrated by the elegant examples from the most recent literature. Some significant progress in the reaction of indirect activation of substrates through in situ generation of numerous supramolecular catalysts from B(C_6_F_5_)_3_ based on Lewis-acid-assisted Lewis acid (LLA) or Lewis acid assisted Brønsted acid (LBA) strategies or the reaction promoted by cooperative actions of chiral co-catalysts and B(C_6_F_5_)_3_ which played a direct role on the activation of substrates have been demonstrated in this review.

## 1. Introduction

Since first illustrated by Gilbert N. Lewis in 1923, the Lewis acids (LAs) and their interactions with Lewis bases (LBs) have been a major and fundamental part of organic chemistry [1]. Various chemical transformations assisted by Lewis acids which encompass the Friedel–Crafts reaction, the Diels–Alder reaction, and the Aldol condensation reaction, are approached as indispensable attributes of synthetic science [2]. Owing to the exceptional particularity in structures, the boron-based compounds are among the most ubiquitous main group Lewis acids, and the borane-based Lewis acids have found a broad implication spanning reagents in organic synthesis, activators for olefin polymerization, organometallic pre-catalysts and particularly as components of frustrated Lewis pairs (FLPs) which was proposed in 2006 by Stephan upon the discovery of reversible activation of H_2_ mediated by a phosphinoborane [1,3,4,5,6].

**Figure 1:**
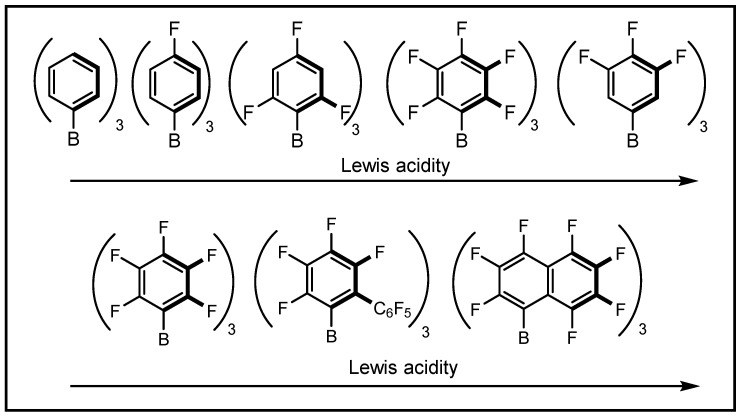
The Lewis acidity of fluoroaryl boranes with different substitutions.

Regarded as an indispensable branch of the borane-based Lewis acids, the fluoroaryl boranes have gained regenerated interest for their characteristic property such as strong Lewis acidity and large steric bulk. Notably, the Lewis acidity and reactivity of fluoroaryl boranes can be regulated through subtle changes in the aryl rings [7]. For example, the number of fluorine atoms around the aryl ring imparted a strong influence on Lewis acidity, with an increased number of fluorines resulting in an increased Lewis acidity [8,9]. The general trend of Lewis acidity for fluorinated arylboranes is shown in Figure 1. Particularly, the B(C_6_F_5_)_3_ has continued to be the focus of intense research efforts because of its unique properties and high reactivity, which have been demonstrated by elegant examples from the most recent literature [10,11,12,13,14].

However, compared with the great attention paid to summarizing the hydrosilylation and hydroboration reactions catalyzed by B(C_6_F_5_)_3_, to the best of our knowledge, there is still no such review devoted to emphasizing the breakthrough on the asymmetric catalysis reactions with the assistance of B(C_6_F_5_)_3_. Therefore, it is timely and desirable to highlight the main developments in this area to attract more research interests to related fields. In this regard, on the basis of our previous works and outcomes, we will summarize the recent progress on the application of B(C_6_F_5_)_3_ in asymmetric catalysis in this review. In the following sections, these related studies will be discussed in detail, mainly based on the two different strategies involving the reaction of indirect activation of substrates through the in situ generation of numerous supramolecular catalysts from B(C_6_F_5_)_3_ based on Lewis-acid-assisted Lewis acid (LLA) or Lewis acid assisted Brønsted acid (LBA) strategies or the reaction promoted by cooperative actions of chiral co-catalysts and B(C_6_F_5_)_3_ which plays a direct role on the activation of substrates.

## 2. Discussion

**Figure 2:**
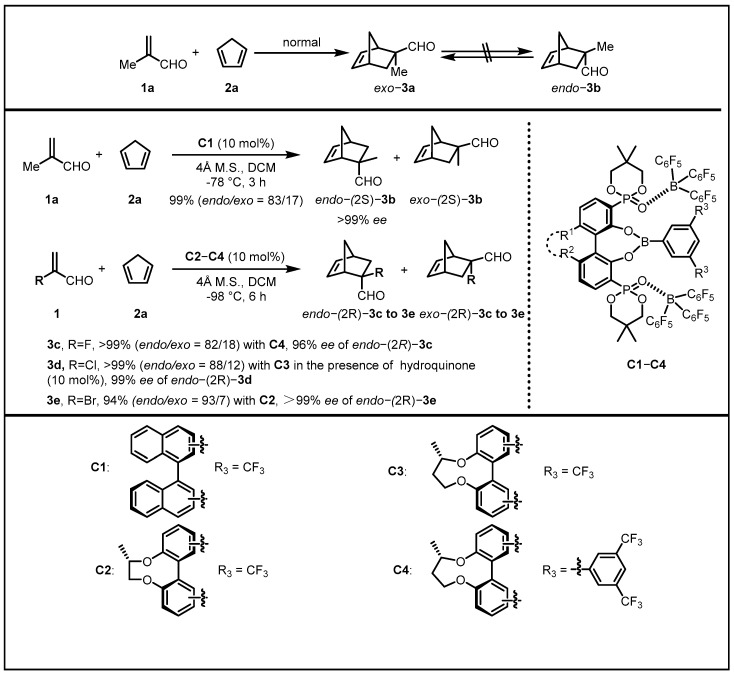
Anomalous *endo*-selective asymmetric reactions between cyclopentadiene and α-substituted acroleins.

Serving as one of the most powerful strategies for the construction of six-membered carbocyclic and heterocyclic systems, the Diels–Alder cycloaddition reaction exhibits prominent synthetic utilities, whereas there is still a widespread perception that the *endo/exo* selectivity in the Diels–Alder reaction strongly depends on the substrates [15,16,17,18]. Consequently, controlling both enantioselectivity and anomalous *endo/exo* selectivity by conventional chiral catalysts remains a long-sought but challenging task for synthetic chemists. For example, in the reaction between cyclopentadiene **2a** and α-substituted acrolein, an *exo* preference may occur on account of the steric interactions between the methylene moiety of cyclopentadiene and the substitutional group R (R≠H) at the α position of acrolein. Moreover, it is infeasible to furnish *endo*-**3b** by the epimerization of *exo*-**3a** for the quaternary carbon center of it (Figure 2) [19].

To address this underdeveloped issue, the Ishihara group developed a series of conformationally flexible chiral supramolecular catalysts (**C1**–**C4**) readily prepared in situ from chiral BINOL-derived phosphoric acids, phenylboronic acid, and B(C_6_F_5_)_3_ [19]. In their design, it was of great necessity for the catalysts to distinguish chiral transition-state structures via accurately recognizing the *re* or *si* face of dienophiles as well as the *endo* or *exo* approach of dienes. In this context, the intermolecular acid–base coordinate bonds in the two P=O···B(C_6_F_5_)_3_ moieties provided a decisive flexibility in conformation accompanied with B(C_6_F_5_)_3_ acting as a bulky functional group to generate a chiral, narrow, and deep cavity, the strong electron-accepting nature of which also increased the Lewis acidity of the central boron through conjugated bonds. Building on this design, they employed the supramolecular catalysts in the Diels–Alder reaction between **1a** and **2a**, giving the anomalous product *endo*-(2S)-**3b** as the major product in 83% yield with 99% *ee*. Aiming to further explore anomalous *endo*-selective Diels–Alder reactions, they examined the reactions between **1a** and α-haloacroleins, which were extremely reactive, thus imposing restrictions on their application on Diels–Alder reactions and normally provided *exo* adducts as major products. After the optimization of the chiral biaryl skeleton, the coworkers successfully established high anomalous *endo/exo* selectivity of Diels–Alder reactions between cyclopentadiene and α-bromoacrolein, α-chloroacrolein, and α-fluoroacrolein in remarkable yields ranging from 82–93% and excellent *ee* (>99%).

**Figure 3:**
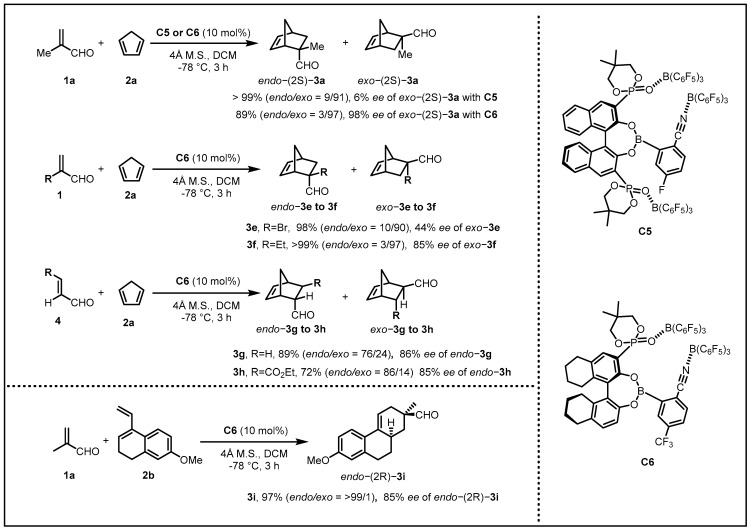
Reactions of various acroleins with cyclopenta-1,3-diene.

Having obtained considerable experience that the two bulky B(C_6_F_5_)_3_ moieties were coordinated to phosphoryl (PO) groups that were essential to the formation of a deep chiral cavity, Ishihara and his coworkers assumed that there existed the possibility that the CN···B(C_6_F_5_)_3_ could serve as another coordination bond to furnish a new type of chiral supramolecular catalysts in light of the CN moiety exerting a steric effect as well as an electron-withdrawing effect on the active boron center. In this regard, Ishihara extended this new kind of supramolecular catalysts on enantioselective Diels–Alder reactions of various acroleins with cyclic or acyclic dienes. Unfortunately, the enantioselectivity toward the product *exo*-(2S)-**3a** was poor with **C5**. After the replacement of the binaphthyl skeleton in **C5** with the octahydrobinaphthyl skeleton and the replacement of the 5-fluoro group in the arylboronic acid moiety with 5-trifluoromethyl to generate catalyst **C6**, the enantioselectivity was distinctly improved to 98% *ee*. (Figure 3) [20].

Next, they investigated the scope of substrates suitable for use with catalysts **C6**. The α-substituted acroleins **3e**, **3f**, **3g**, and **3h** were examined to be effective. The effect of replacing cyclopenta-1,3-diene with acyclic diene was also examined, resulting in **3i** exclusively in 97% yield and 85% *ee*. Unlike the cases with their previous **C1**–**C4**, in the Diels–Alder reactions of **3e** and **3f** in the presence of **C6**, extraordinary *endo*/*exo*-selectivities were not observed. The structure of **C6** might be too flexible to perform well in control of excellent *endo/exo*-selectivities. Instead, with more flexibility, the **C6** induced high enantioselectivities with a relatively wide range of substrates. Having conducted fundamental control experiments, they found that the CN···B(C_6_F_5_)_3_ moiety, as well as an electron-withdrawing group such as trifluoromethyl in **C6** was essential for obtaining the Diels–Alder products in high yields and high enantioselectivities.

**Figure 4:**
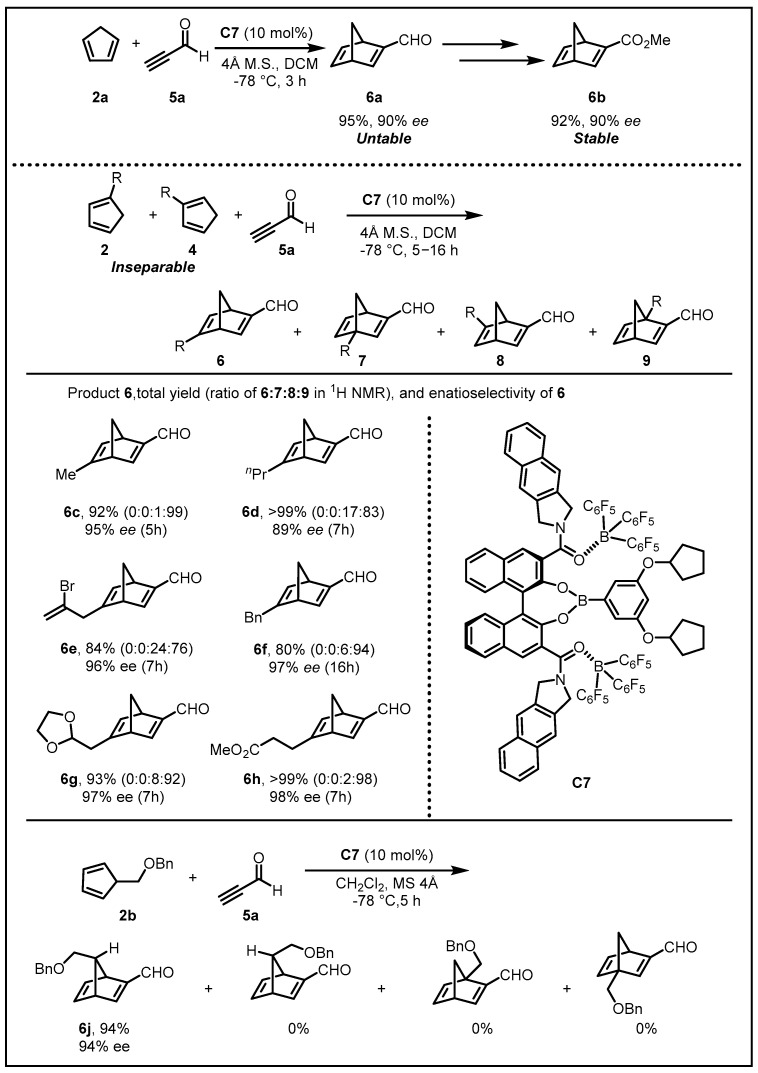
LLA-induced enantioselective Diels–Alder reactions with high multiselectivities.

With only one prior study focusing on the reaction of propargyl aldehyde **5a** with cyclopentadiene **2a** with a chiral boron Lewis acid catalyst reported by Yamamoto [21] in low yield of **6a** due to its overreaction with **2a**, Ishihara’s group were currently interested in whether or not their chiral supramolecular U-shaped catalysts could control multiselectivities in such reactions. After thorough optimization, the catalyst **C7** was found to be the most effective in the reactions of **2a** with **5a** to afford **6a** in 95% yield with 90% *ee.* It was noteworthy that the **6a** could be transformed into synthetically useful optically active compounds such as α,β-unsaturated ester **6b**, which was more stable and would be more suitable for 1,2- and 1,4-addition reactions with some nucleophiles to give various high-value products such as Corey’s chiral diene ligand for asymmetric transition-metal catalysis or the key intermediate of (+)-Sarkomycin (Figure 4) [22].

In order to probe the substrate- and regioselectivity, the DA reactions between **5a** and 1-/2-alkyl-substituted cyclopentadienes **2/4** were carried out, which could not be isolated from a 1:1 mixture due to isomerization. Generally, if in bad regioselectivity, eight-isomeric first DA adducts and numerous (theoretically, up to 64) corresponding second DA adducts would be delivered in principle. Delightedly, high substrate-, enantio-, *endo/exo*-, and regioselectivities were obtained with catalyst **C7**, giving the exclusively desired DA adducts in a yield up to 92% with 95% *ee*. Moreover, the π-facial-selective DA reaction of 5-alkylsubstituted cyclopentadiene **2b** was also examined, giving the exclusively corresponding product **6j** in 94% yield with 94% *ee* with the use of **C7**, showing remarkable π-facial selectivity as well as enantio-, *endo/exo*-, and substrate-selectivities.

**Figure 5:**
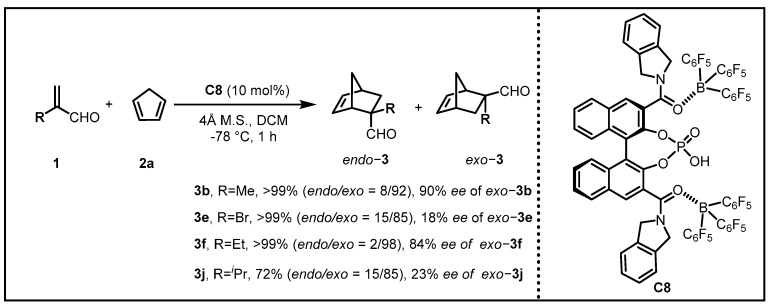
Substrate specificity with the use of LBA catalyst **C8**.

After great progress was made in the supramolecular catalysts based on the Lewis acid assisted Lewis acid (LLA), the team envisioned that it seemed feasible to develop a conformationally flexible chiral supramolecular Brønsted acid catalyst based on the Lewis acid assisted Brønsted acid (LBA) catalyst system for asymmetric catalysis. In consideration of the bifunctional role of chiral phosphoric acids serving as both Brønsted acid and Brønsted base, the acroleins might be capable of doubly coordinating with active centers, contributing to the enantioselective Diels–Alder reactions with cyclopentadiene. After the enantioselectivity was enhanced to 90% *ee* in the presence of chiral supramolecular catalysts **C8**, they examined the substrate specificity for α-substituted acroleins (Figure 5) [23]. Unfortunately, except for the probe reaction of methacrolein **3b** together with ethylacrolein **3f**, the other acrolein, such as α-isopropylacrolein **3j** as well as α-bromoacrolein **3e**, only gave the corresponding *exo* products in low enantioselectivities, partially due to steric mismatch with the chiral cavity of **C8**. However, in spite of the limited scope of the substrates, this strategy opened a new venue for designing supramolecular catalysts containing a conformationally flexible, bulky, and chiral cavity for higher-ordered catalysis.

**Figure 6:**
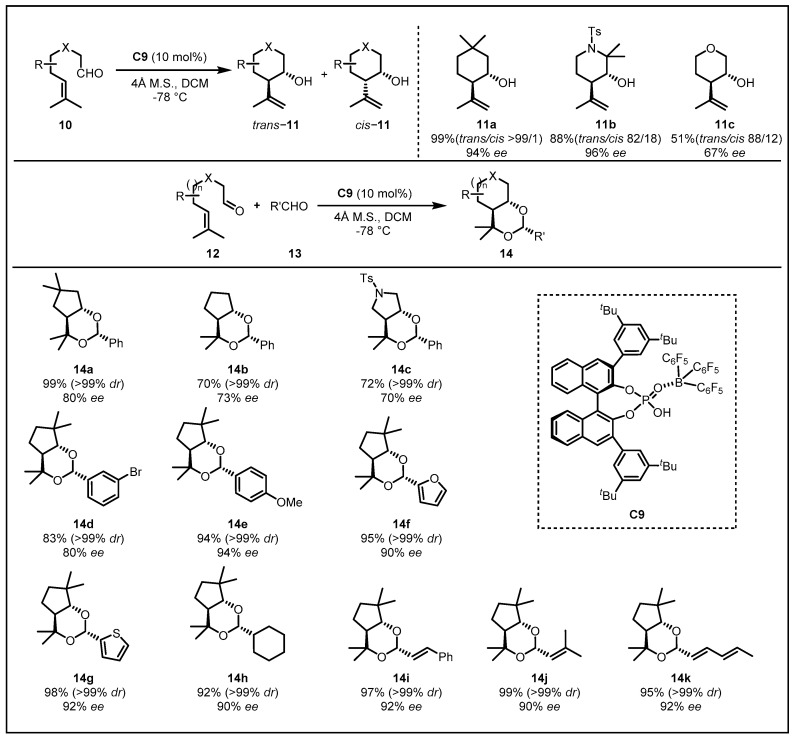
Carbonyl-ene cyclization and carbonyl-ene cyclization–acetalization tandem reactions catalyzed by **C9**.

Much later, greatly encouraged by List’s report [24], which illustrated the first organocatalytic enantioselective carbonyl-ene cyclization with confined chiral Brønsted acid catalysts based on a BINOL-derived imidodiphosphate and on the consideration of their success on the LBA induced enantioselective Diels−Alder, [2+2] cycloaddition, and hetero-Diels−Alder reactions [25,26], they extended the probability of LBA catalyst system in such carbonyl-ene cyclization. Initially, to confirm the optimal choice of the catalysts, they examined the carbonyl-ene cyclization reaction with the combination of B(C_6_F_5_)_3_ and (R)-3,3′-Ar_2_-BINOL-derived phosphoric acid catalyst. To their delight, the **C9** was be proved to the best through the screening of the 3,3′-substituents of the BINOL structure, inducing **11a** in a high enantioselectivity (94% *ee*), a good diastereoselectivity and yield (>99%) (Figure 6) [27]. With the optimized reaction conditions in hand, they examined a variety of typical non-substituted alkenyl aldehydes, which was still challenging in the carbonyl-ene cyclization because of the absence of the Thorpe−Ingold effect. As a result, the corresponding tetrahydropyran and cyclohexane **14a**–**14c** were obtained with a high *trans/cis* ratio and a satisfactory yield as well as enantioselectivity. Furthermore, an unexpected carbonyl-ene cyclization–acetalization tandem reaction was realized with the addition of aldehydes. Both aromatic aldehydes with electron-withdrawing, as well as electron-donating groups and heteroaromatic aldehydes, could be tolerated, revealing moderate to high enantioselectivities (**14d**–**14k**, 80–94% *ee*) and diastereoselectivities (>99% *dr*) for the first time. For aliphatic cyclohexane, carboxaldehyde with a branched alkyl moiety also offered the corresponding product **14h** with >99% *dr* and 90% *ee*. Generally, there occurred an undesired tendency for α,β-unsaturated aldehydes to take Michael’s addition of the alcohols when performing the selective acetalization, which was hard to control. In sharp contrast, a sequence of α,β-unsaturated aldehydes could also be used in this strategy, and the corresponding acetals **14i**–**14j** were obtained in 97–99% yields with >99% *dr* and 90–92% *ee*. It was noteworthy that α,β,γ,δ-unsaturated aldehyde could also be employed, and the product **14k** was obtained in 95% yield with >99% *dr* and 92% *ee*.

The usage of such supramolecular catalysts pioneered by Ishihara and coworkers has attracted considerable attention in the scientific community. Although subsequent development made by the Du group [28,29,30] and Wang group [31,32,33,34,35] has greatly extended the scope of such chiral Lewis acidic boranes, however, there remains a fundamental challenge, that is, its nonnegligible limitations on the protocols for the synthesis of them. On the other hand, the cooperation of B(C_6_F_5_)_3_ with chiral ligands or chiral catalysts to realize asymmetric catalytic reactions has been an emerging field that distinctly avoids such problems. In this regard, the Wasa group developed various strategies by employing enantioselective cooperation of Lewis acidic B(C_6_F_5_)_3_ and Brønsted basic N-based electrophiles, which has realized the direct conversion of α-C–H bonds contained in N-alkylamines into α-C–alkynyl bonds, which is an attracting way for the pharmaceutical industry.

**Figure 7:**
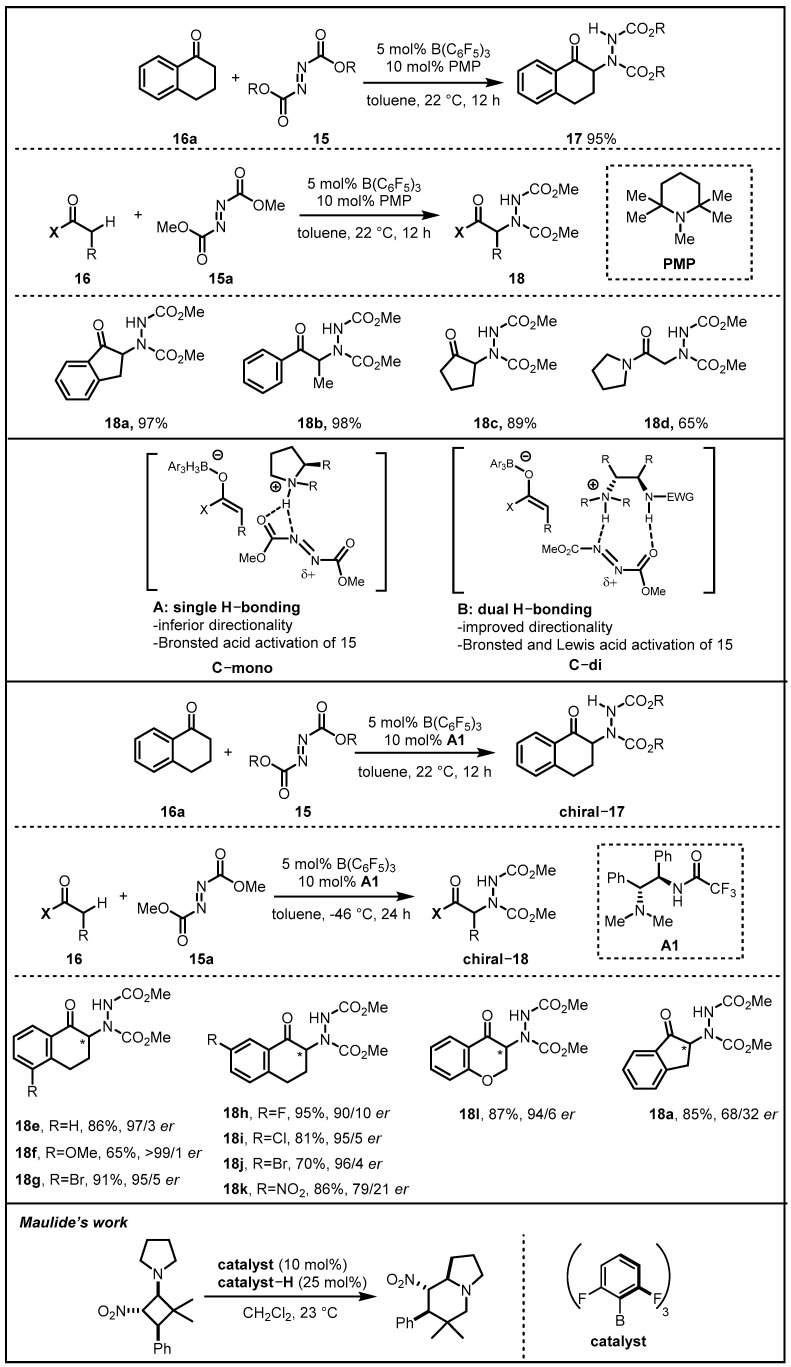
B(C_6_F_5_)_3_/amine-catalyzed direct α-amination reactions.

In 2016, they envisioned that the acidity of an α-C–H bond in carbonyl pronucleophiles could be enhanced by the participation of a boron-based Lewis acid, which was followed by deprotonation by a hindered amine, resulting in the formation of a tightly bound ionic pair encompassing a boron enolate and an ammonium cation. Due to the Brønsted acidity of the ammonium cation, it might contribute to the activation of electrophilic amination reagent while exactly positioning it for reaction with the enolate component to offer aminocarbonyl products. A noteworthy superiority of the strategy was that it was not imperative to tether acidic and basic catalyst components, resulting in the feasibility of facile and independent modification of each component for optimization of reaction efficiency or enantioselectivity. A preliminary examination was conducted to explore the ability of achiral Lewis acid/Brønsted base catalysts to promote the desired transformation. α-tetralone **16a** and dialkyl azodicarboxylates **15** were reacted with B(C_6_F_5_)_3_/amines serving as potential catalysts, and the transformation proceeded efficiently when PMP was employed (Figure 7) [36]. Alkyl-substituents of azodicarboxylate were found to have a better performance in the reaction. With the optimized reaction conditions in hand, they then focused on the development of an enantioselective version of the catalytic process with **16a** serving as the model substrate. Having tried the tertiary amine moiety of **C-mono**, which was expected to play the role of a Brønsted base in deprotonation of B(C_6_F_5_)_3_-activated **16a** and subsequently transformed into a Brønsted acid that could associate with a basic moiety of **16a** by a single H-bonding interaction, undesirable enantioselectivity was obtained. Therefore, Wasa and coworkers presumed that it was insufficient for the single H-bonding interaction between the chiral ammonium ion and **16a** to induce a highly enantioselective C−N bond-forming process. Consequently, they changed to evaluate the dual H-bond donors derived from the amine groups of **C-di** for the fact that dual H-bonding interactions could deliver additional electrophile activation, which accelerated the formation of enantioselective C–N bond via enhancing conformational restriction. Satisfyingly, the more electron-withdrawing and less hindered catalyst **A1** was selected to be the most effective, and a range of cyclic ketones were suitable for enantioselective direct α-amination reactions catalyzed by 5 mol% B(C_6_F_5_)_3_ and 10 mol% **A1**. Methoxy, fluoro-, chloro-, and bromo-substituted α-tetralone derivatives were smoothly converted to the corresponding products in 90:10 to >99:1 *er*. Notably, by means of similar design, the Maulide group realized a variety of alkaloid-like products formed in an enantio- and diastereoselective manner through the combination of chiral anime and achiral Lewis organoborane acid in 2022 [37].

**Figure 8:**
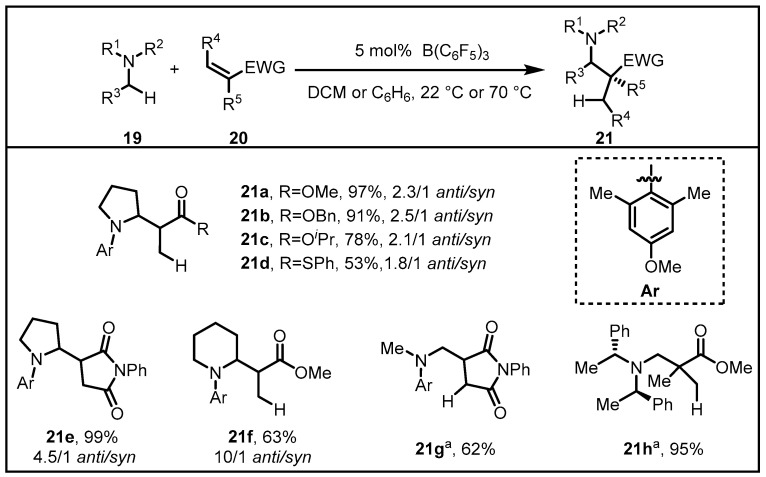
Coupling of N-alkylamines and α,β-unsaturated compounds through B(C_6_F_5_)_3_-catalyzed hydride abstraction.

On the other hand, in the design of a catalytic process for stereoselective coupling of N-alkylamines and carbon-based nucleophiles, the chiral anime might not be indispensable. The combination of B(C_6_F_5_)_3_ and a chiral Lewis acid co-catalyst would also work well in this aspect [38]. B(C_6_F_5_)_3_ could abstract a hydride from amine, thus forming a borohydride along with an iminium ion. In the meantime, a chiral Lewis acid co-catalyst would activate the α,β-unsaturated substrate to promote reduction by the borohydride, affording the corresponding chiral enolate. Followed by the stereoselective reaction between the iminium ion and the chiral enolate, the resulting α,β-amino carbonyl product would be generated. In light of this design, the Wasa group assumed it was essential that an appropriate combination of an acidic catalyst and an amine substrate should be confirmed to allow for efficient hydride abstraction, finding that highly acidic B(C_6_F_5_)_3_ together with sterically demanding and electron-rich N-alkylamines represented the most effective combination (Figure 8) [39]. Ultimately, a variety of α,β-unsaturated carbonyl compounds, and related electrophiles were used in the reaction with N-arylpyrrolidine to generate the corresponding α-substituted amines in excellent yields.

**Figure 9:**
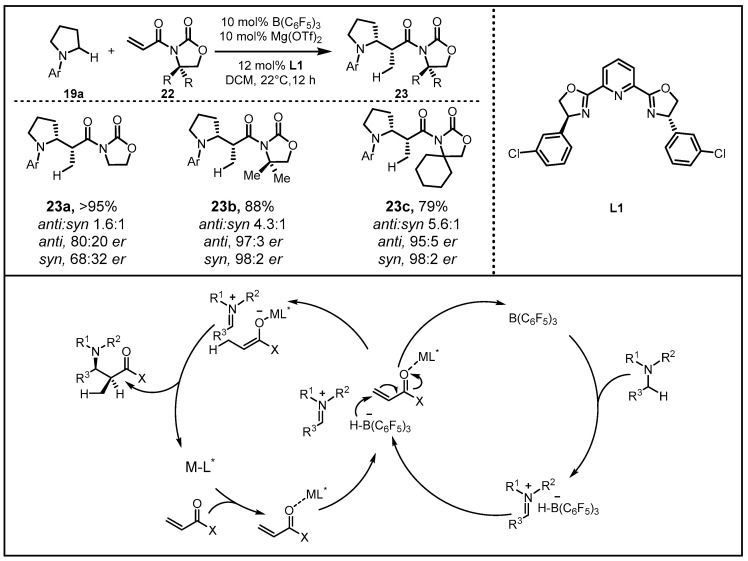
B(C_6_F_5_)_3_ and Mg(OTf)_2_/**L1**-induced synthesis of β-amino amides.

After the success of hydride abstraction through single-catalyst racemic transformation, they further developed an enantioselective version of the catalytic process by employing B(C_6_F_5_)_3_ in cooperation with a matched chiral Lewis acid. With N-arylpyrrolidine and 3-acryloyloxazolidin-2-one as model substrates, systematic evaluation of Lewis acid/chiral ligand complexes was carried out, suggesting that B(C_6_F_5_)_3_ and Mg(OTf)_2_/**L1** serves as optimal catalysts. Subsequently, they surveyed a series of achiral and chiral N-acryloyloxazolidinones, indicating that the two-catalyst protocol was applicable to the synthesis of a variety of N-alkylamines, affording corresponding products as separable mixtures of diastereomers generated in >99:1 *er* in either isomeric form (Figure 9) [39].

**Figure 10:**
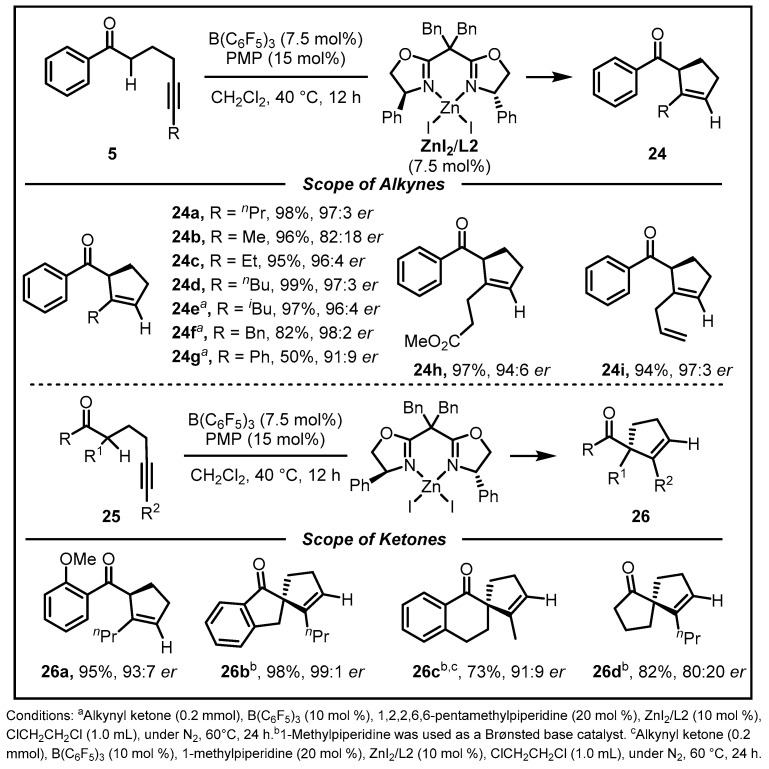
Enantioselective Conia-Ene-type cyclizations of alkynyl ketones.

One year later, they further extended such synergetic catalyst systems in facilitating direct enantioselective Conia-ene-type processes with mono-carbonyl compounds by the collaboration of B(C_6_F_5_)_3_, N-alkylamine and a chiral Lewis acid co-catalyst. In their envision, the ability of B(C_6_F_5_)_3_ might be able to be controlled to function as an activator of a carbonyl along with elevating the reactivity of alkyne in the presence of chiral Lewis acid co-catalyst. Then, an enolate and an ammonium ion were generated via deprotonation of B(C_6_F_5_)_3_-activated ketone with the assistance of N-alkylamine. Concurrently, a chiral Lewis acid co-catalyst (ML*) would activate the alkyne units. An ensuing enantio-determining 5-*endo*-*dig* cyclization of the enolate and the alkyne would deliver a key intermediate, which underwent subsequent protonation of the C−ML* bond of itself by the ammonium ion, providing the desired cyclopentenyl products. In order to realize this catalytic cycle effectively, an optimal catalyst combination was conducted, demonstrating that the B(C_6_F_5_)_3_, sizable and electron-rich PMP together with sterically demanding ZnI_2_/**L2** complex emerged as the combination with the best efficiency. A variety of 1-phenyl ketones with different alkyne substituents and different aryl- and alkyl-substituted ketones proved to be suitable substrates. Reactions involving substrates that carried ethyl, *n*-propyl, *n*-butyl, and *iso*-butyl substituent, afforded the desired products in >95% yield and 96:4–97:3 *er* (Figure 10) [40].

**Figure 11:**
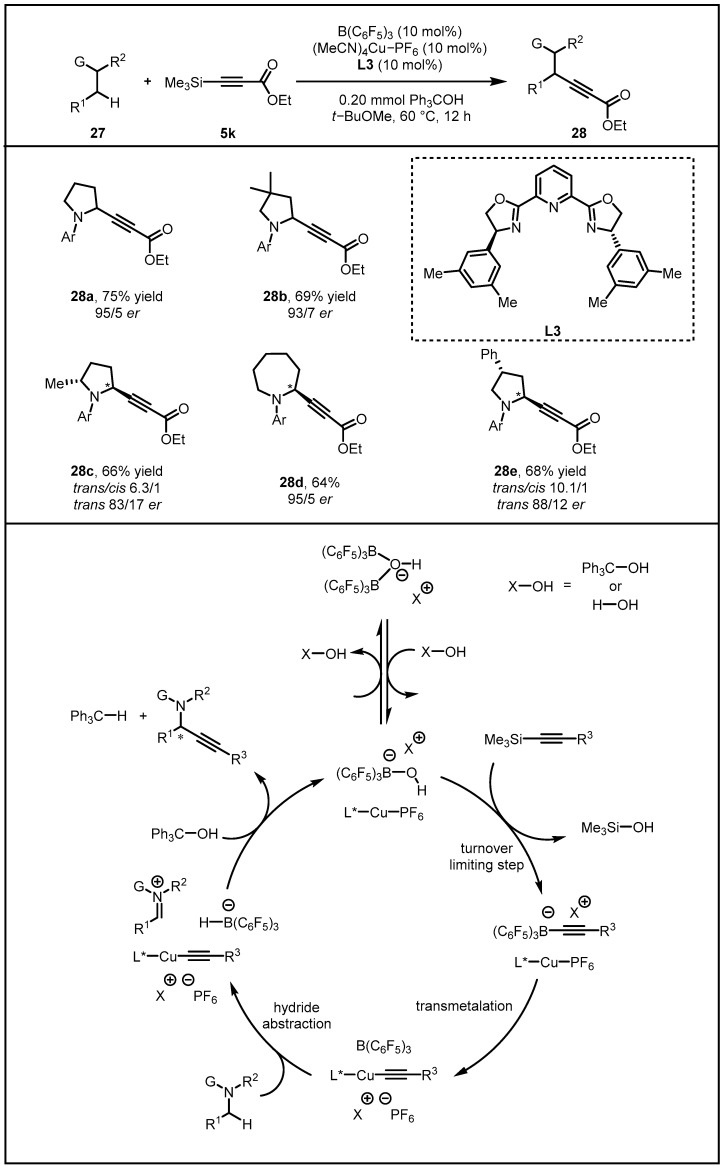
Direct conversion of N-Alkylamines to N-propargylamines promoted by Lewis acid/organocopper catalysis.

Showing that by tuning of different features of Lewis acids that possess overlapping functions, it was possible to engage Lewis acid catalysts to serve as an activator of a carbonyl group or an activator of electron-rich alkyne, and they next attempted to apply such strategy in entailing the conversion of an α-amino C(sp^3^)–H bond of N-alkylamines into an α-C–alkynyl bond. Fortunately, after probing the ability of B(C_6_F_5_)_3_ and various Cu-based complexes to catalyze the reaction, the (MeCN)_4_CuPF_6_/**L3** complex with Ph_3_COH as an additive was determined to function well in developing a stereoselective version of the catalytic C–alkynyl bond forming. Meanwhile, reactions with an array of N-alkylamines were carried out under optimal conditions. N-arylpyrrolidines as well as N-arylazepane bearing the α-alkynyl group were thus synthesized in 64–75% yield and 83:17–95:5 *er*. Additionally, a range of enantiomerically enriched pyrrolidine substrates underwent transformation occurring at the less hindered α-amino C–H bond. To shed light on the mechanism of the catalytic process, and series of investigations were carried out, illustrating that B(C_6_F_5_)_3_ might receive a hydride from an amine, generating a borohydride and an iminium ion. Subsequently, a Cu-based catalyst might undergo transmetalation with alkynylsilane with the aid of an alcohol additive to afford a L_n_Cu–alkynyl complex (II) and trimethylsilanol. An ensuing C–C bond formation between in situ generated L_n_Cu–alkynyl complex and iminium ion would afford the desired propargylamines. Hydride transfer from borohydride to R–OH-derived cationic species would then regenerate B(C_6_F_5_)_3_, thereby closing the cycle (Figure 11) [41].

**Figure 12:**
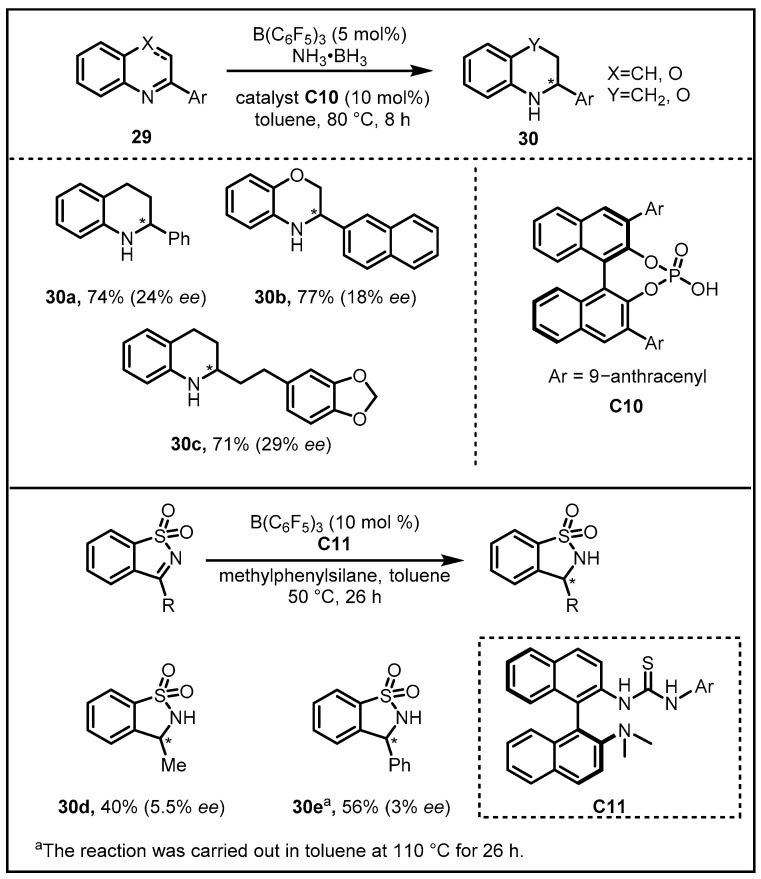
Asymmetric transfer hydrogenations of quinolines and indole derivatives.

It is notable that the hydrogenations of N-heterocycles with ammonia borane, particularly the hydrogenations of quinoline and indole derivatives, have not been disclosed before the work reported by our group. With the assistance of B(C_6_F_5_)_3_, a new strategy promoting hydrogenations of N-heterocycles with ammonia borane under transition-metal-free conditions has been recently realized. In spite of low enantioselectivity, the results preliminarily presented by our group confirmed the feasibility and potential and paved the way for the application of B(C_6_F_5_)_3_ in the such field (Figure 12) [42,43].

## 3. Conclusions

In conclusion, we have summarized some significant progress in the reaction of indirect activation of substrates through the in situ generation of numerous supramolecular catalysts from B(C_6_F_5_)_3_ based on Lewis-acid-assisted Lewis acid (LLA) or Lewis acid assisted Brønsted acid (LBA) strategies or the reaction promoted by cooperative actions of chiral co-catalysts and B(C_6_F_5_)_3_ which plays a direct role on the activation of substrates. With these promising advances, it can be expected that further advancements of B(C_6_F_5_)_3_-catalyzed asymmetric reactions to be realized by electrochemistry and photochemistry will be obtained in the near future.

## Data Availability

Not applicable.
